# Beyond destruction: emerging roles of the E3 ubiquitin ligase Hakai

**DOI:** 10.1186/s11658-025-00693-y

**Published:** 2025-01-20

**Authors:** Juan-José Escuder-Rodríguez, Andrea Rodríguez-Alonso, Lía Jove, Macarena Quiroga, Gloria Alfonsín, Angélica Figueroa

**Affiliations:** Epithelial Plasticity and Metastasis Group, Instituto de Investigación Biomédica de A Coruña (INIBIC), Complexo Hospitalario Universitario de A Coruña (CHUAC), Sergas, Universidade da Coruña (UDC), Xubias de Arriba 84, 15006 A Coruña, Spain

**Keywords:** Hakai, *CBLL1*, E3 ubiquitin ligase, m^6^A methyltransferase complex, Cancer, Targeted therapy, Prognostic biomarker

## Abstract

Hakai protein (*CBLL1* gene) was identified as an E3 ubiquitin ligase of E-cadherin complex, inducing its ubiquitination and degradation, thus inducing epithelial-to-mesenchymal transition. Most of the knowledge about the protein was associated to its E3 ubiquitin ligase canonical role. However, important recent published research has highlighted the noncanonical role of Hakai, independent of its E3 ubiquitin ligase activity, underscoring its involvement in the *N*^6^-methyladenosine (m^6^A) writer complex and its impact on the methylation of RNA. The involvement of Hakai in this mRNA modification process has renewed the relevance of this protein as an important contributor in cancer. Moreover, Hakai potential as a cancer biomarker and its prognostic value in malignant disease also emphasize its untapped potential in precision medicine, which would also be discussed in detail in our review. The development of the first small-molecule inhibitor that targets its atypical substrate binding domain is a promising step that could eventually lead to patient benefit, and we would cover its discovery and ongoing efforts toward its use in clinic.

**Graphical Abstract:**

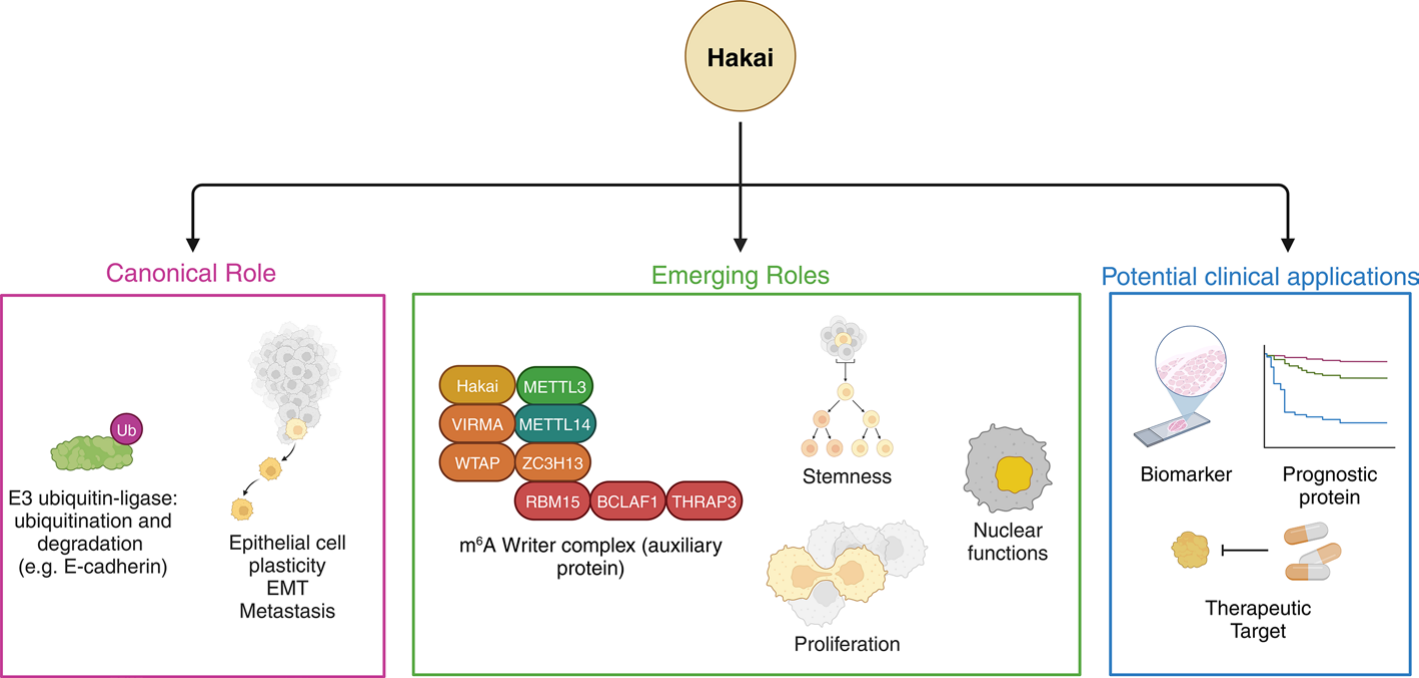

## Introduction

Hakai [Cbl proto-oncogene-like 1 (CBLL1) gene] protein was first identified as a specific E3 ubiquitin ligase for E-cadherin [1], and its name, derived from the Japanese word for “destruction,” reflects its role in ubiquitinating and subsequently degrading the E-cadherin complex. Ubiquitination is the second most common post-translational modification after phosphorylation, and it plays a role in regulating protein stability, interactions, and activity. During the ubiquitination process, specific target proteins are tagged by ubiquitin, a small 76 amino-acid-long protein [2]. Ubiquitination not only leads to degradation by the proteasome, lysosomes, or autophagy but also regulates other processes such as transcription, DNA repair, or protein localization [3]. The ubiquitination system operates as an enzymatic cascade consisting of an activating enzyme (E1), a conjugating enzyme (E2), and a ligase enzyme (E3). E3 ubiquitin ligase enzymes are responsible for substrate specificity, making them more desirable targets for drug targeting than are the E1-activating and E2-conjugating enzymes. Ubiquitination is a reversible process regulated by deubiquitinases (DUBs), proteases that remove ubiquitin chains to modulate its biological functions [4].

**Fig. 1:**
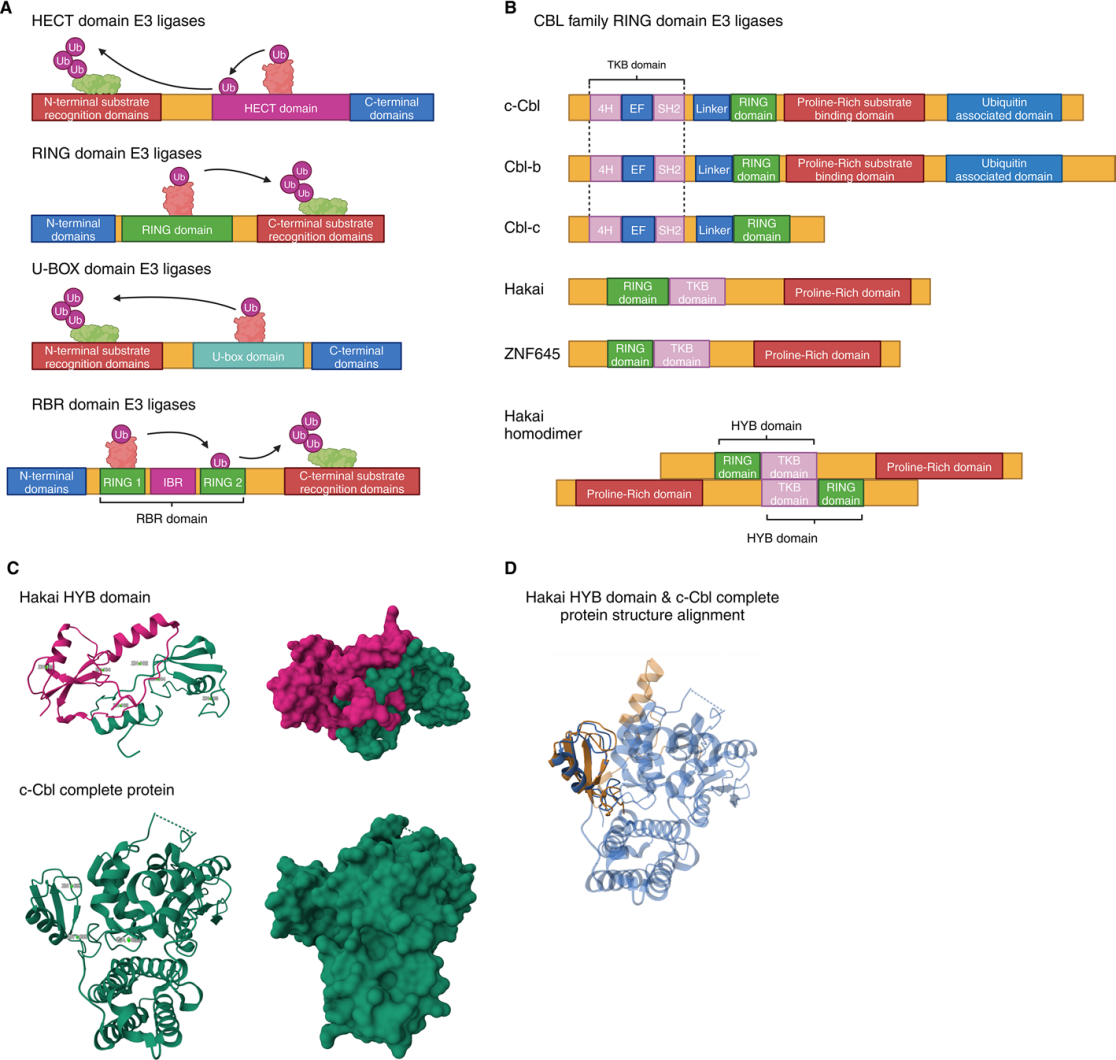
**A** Classification of E3 ubiquitin ligases by domain architecture. The substrate protein (green) and E2 conjugating enzyme (red) may bind different regions of the enzyme. In HECT and RBR E3 ligases, ubiquitin is transferred to the E3 enzyme, while RING and U-Box ligases directly transfer ubiquitin to the substrate. **B** The Cbl family of RING E3 ubiquitin ligases. The N-terminal tyrosine kinase binding (TKB) domain contains four-helix bundles (4H), an EF hand (EF), and a Src homology (SH2) domain. This is followed by the RING domain, responsible for E3 activity, and the proline-rich domain for substrate recognition. The C-terminal ubiquitin-associated domain (UAB) facilitates ubiquitin binding and dimerization. Cbl-c lacks both the proline-rich and UAB domains, whereas c-Cbl and Cbl-b contain all domains. Hakai has an inverted domain order, and its RING domain is near the N-terminus, followed by the PTB domain and lacking the UAB domain. ZNF645 shares high homology and similar domain architecture with Hakai but is shorter. Both form an atypical pocket called the Hakai phospho-tyrosine binding (HYB) domain upon homodimerization, involving the RING and PTB domains. The HYB domain in Hakai dictates substrate specificity and is a potential drug target. **C** Comparison of Hakai HYB domain dimer PDBID 3VK6 [13, 111] and c-Cbl PDBID 2Y1M [112, 113] structures. The Hakai HYB domain dimer structure, with zinc coordination from both monomers (green and pink), is unique to Hakai and absent in other RING E3 ligases. **D** Structure alignment of HYB domain (orange) and c-Cbl (blue). The overlapping region corresponds to the RING domains (left). The structures were retrieved from the protein databank [114], (http://www.rcsb.org/) and were created with BioRender.com

E3 ubiquitin ligases are crucial for substrate specificity in the ubiquitination system. The human genome encodes over 600 E3 ubiquitin ligases but only two E1 ubiquitin-activating enzymes [5] and approximately 32 E2 ubiquitin-conjugating enzymes [6]. E3 ubiquitin ligases have been classified according to their catalytic domain and the mechanisms they use to transfer ubiquitin to target proteins (Fig. 1A). The classification includes really interesting new gene (RING)-type domain proteins and homologous to E6-associated protein carboxyl terminus (HECT) domain proteins [7]. Other less frequent types have also been described, including U-box domain proteins [8] and RBR (RING-between-RING) domain proteins [9]. RING domain E3 ubiquitin ligases are the most abundant with an estimated of 270 genes in humans [10]. RING domain ubiquitin ligases facilitate the direct transfer of ubiquitin from the E2 enzyme to the protein substrate via their RING domain [11] and can function as single-chain enzymes, homodimers, heterodimers, or as part of multisubunit complexes. Some RING ligases, including the casitas B-lineage lymphoma (Cbl) family and Hakai, recognize the specific substrates in a phospho-tyrosine (pTyr)-dependent manner. The Cbl family consists of three members in mammals, Cbl (also known as c-Cbl), Cbl-b, and Cbl-c (also known as Cbl-3) [12].

Hakai is a 491 amino acid RING-type domain E3 ubiquitin ligase and resembles the Cbl family, with a similar structure to that of c-Cbl [1]. However, Hakai is not a typical Cbl protein. Similar to the Cbl family members, Hakai contains a phosphotyrosine-binding domain (PTB), a RING finder domain, and a proline-rich domain (Fig. 1B). Nevertheless, the distribution of Hakai domains along the protein sequence is different from the other Cbl family members. Indeed, the RING domain and the PTB domain are in reverse linear order, and the proteins have no sequence similarities outside these domains (Fig. 1B). Furthermore, the binding mechanism to tyrosine-phosphorylated substrates significantly differs between Hakai and c-Cbl. The protein Hakai contains two monomers arranged in an antiparallel manner [13]. Each monomer comprises an N-terminal RING domain and a C-terminal PTB domain that incorporates a zinc coordination motif, which is crucial for dimerization [13]. Hakai dimerization is formed through the binding of pTyr residues of the Hakai monomers in a zinc-coordinated manner, allowing the formation of a phosphotyrosine-binding pocket, named Hakai-phosphotyrosine-binding (HYB) domain [13, 14], which recognizes specific tyrosine-phosphorylated substrates. This binding pocket structurally differs from the binding domains of other RING family E3 ubiquitin ligases (Fig. 1C-D). Unlike other E3 ubiquitin ligases that do act as dimers, Hakai dimerization does not occur through RING domain association, and instead, it is mediated by its pTyr peptide [14]. So far, in addition to Hakai (*CBLL1* gene), the HYB domain has been found only in ZNF645 (*CBLL2* gene), exclusively expressed in normal human testicular tissue [15].

E-cadherin is the most established component of the adherent junctions at cell‒cell contacts. The loss of E-cadherin induced by Hakai, upon phosphorylation by the protein kinase Src, leads to its endocytosis, disrupting cell‒cell contacts and, subsequently, inducing the epithelial-to-mesenchymal (EMT) transition process, a critical process involved in cell invasion, tumor progression, and metastasis [16, 17]. Moreover, Hakai expression is increased in several types of cancer tissues, including colon [18–20], gastric [18], and non‐small cell lung cancer (NSCLC) tissues [21] compared with adjacent nontumor tissues. Considering Hakai mechanism of action, its higher expression in cancer and the structurally unique HYB domain, it has been proposed as a promising therapeutic target for cancer treatment. The fact that the dimerization is essential for Hakai activity as an E3 ubiquitin ligase suggests that allosteric inhibitors targeting the HYB domain have potential therapeutic implications for tumor treatment [14]. Furthermore, the involvement of Hakai in the RNA metabolism has been recently identified, opening a new and critical area of research, which will be explored in depth in this review.

### Role of Hakai as an E3 ubiquitin ligase of E-cadherin

#### E-cadherin, the first reported substrate for Hakai

E-cadherin is a key component of adherens junctions, forming calcium-mediated interactions between two E-cadherin extracellular domains at cell‒cell contacts, with a cytoplasmic domain that interacts with the cell actin cytoskeleton through proteins such as p120-catenin, β-catenin, and α-catenin [22]. Epithelial monolayers are dynamic, and the endosomal recycling of E-cadherin in the plasma membrane plays an important role in this mechanism of cell contact remodeling [23]. Hakai recognizes the cytoplasmic domain of E-cadherin dependent of Src phosphorylation. The cytoplasmic domain of E-cadherin contains two domains known as cadherin homology 2 and 3 (CH2 and CH3). The CH2 domain of E-cadherin includes three tyrosine residues, two of which are specific of this protein and not shared with other cadherins, such as neural cadherin (N-cadherin) and OB-cadherin. The interaction between E-cadherin and Hakai through these two specific residues (pTyr755 and pTyr756 in mice and pTyr753 and pTyr754 in humans) demonstrate the specificity of Hakai to interact with E-cadherin but not to other cadherins or receptor tyrosine kinases [1]. Furthermore, the degradation of E-cadherin by Hakai-mediated ubiquitination is via the lysosome, involving the action of Rab5 and Rab7 GTPases, while the recycling of E-cadherin through Rab11-containing recycling endosomes (RE) to the cell membrane is reduced [23, 24].

E-cadherin loss at cell–cell contacts is probably the best-established hallmark of EMT. The EMT process is characterized by the acquisition of mesenchymal characteristics and the loss of epithelial markers, including E-cadherin. In fact, upregulation of N-cadherin (a mesenchymal marker) and downregulation of E-cadherin are hallmarks of EMT, known as the cadherin switch. The cadherin switch is associated with increased migration and invasion in cancers. E-cadherin also regulates the inhibition of cell proliferation via cell–cell contact inhibition, regulating the expression of receptor tyrosine kinases and tyrosine kinase Src [25]. The extracellular domain of E-cadherin has also been shown to interact with receptor tyrosine kinases such as epithelial growth factor receptor (EGFR) suppressing its proliferation-stimulating signaling [26].

#### Molecular mechanisms involved in the regulation of E-cadherin by Hakai

Owing to the important role of E-cadherin at cell‒cell contacts, controlling the expression of its encoding gene, *CDH1*, has also been reported to have important implications for both development and cancer. The main regulators of *CDH1* are the transcription factors ZEB1, ZEB2, SNAIL, SLUG, and TWIST [26, 27]. However, in recent years, the posttranslational control of E-cadherin by ubiquitination has been highlighted. In fact, it is proposed that the loss of E-cadherin at the first stages of EMT is governed mainly by posttranslational mechanisms that include ubiquitination, endocytosis, and lysosomal degradation, whereas the transcriptional downregulation of *CDH1* plays a role in the later stages of EMT [28]. This suggests that targeting this post-translational event may offer a therapeutic window to prevent or reverse EMT in cancer metastasis before more permanent transcriptional changes occur. With respect to the control of Hakai expression, transforming growth factor beta 1 (TGFB1) induces the transcription of *CBLL1*. Moreover, hyperactivation of Raf, which indirectly enhances E-cadherin tyrosine phosphorylation via the Raf/MAPK pathway, cooperates with an increased ubiquitination of E-cadherin through TGFB1 induction for the degradation of E-cadherin in the early stages of EMT [28].

When E-cadherin is not phosphorylated by Src, Numb protein binds through its PTB domain to a conserved amino acid region, promoting E-cadherin localization in epithelial cells to its lateral domain in cell‒cell contacts. Upon phosphorylation by Src, Hakai binds to E-cadherin in the same conserved region and promotes ubiquitination and endocytosis [29]. Although the specific ubiquitin chain linked has not yet been characterized, K48 and K63 are the lysine residues on ubiquitin that can be linked together to form polyubiquitin chains. Whether an ubiquitinated protein is directed toward proteasomal degradation or sent to the endosomal–lysosomal pathway depends on the specificity of these proteins for K48- or K63-ubiquitin chains [30]. K48 is the most abundant ubiquitin linkage and targets its substrates for proteasomal degradation [31]. Ubiquitinated E-cadherin degradation is a two-step process in which a 90 kDa fragment is first formed by partial degradation in the proteasome [32], after which it is completely degraded into lysosomes [23].

Finally, deubiquitination is also important in the regulation of E-cadherin stability as a counter mechanism for Hakai ubiquitination. The ubiquitin-specific protease 47 (USP47), a DUB enzyme, is transported to adherens junctions by kinesin family member C3 (KIFC3), reversing the ubiquitination of E-cadherin by Hakai [32]. KIFC3 is a minus-end-director motor that migrates to adherens junctions in a calmodulin-regulated spectrin-associated protein 3 (CAMSAP3)-dependent manner. Moreover, the localization of CAMSAP3 to adherens junctions depends on the complex of E-cadherin with p120-catenin and pleckstin homology domain-containing family A member 7 (PLEKHA7). Thus, the stabilizing role of p120-catenin on E-cadherin might also be explained by its involvement in directing the KIFC3–USP47 complex to adheren junctions [32]. Loss of either KIFC3 or USP47 results on increased ubiquitination of E-cadherin and its targeting for proteasomal degradation [32].

#### Ajuba, cortactin, and DOK1 as novel Hakai substrates

Identifying novel substrates for E3 ubiquitin ligases is challenging due to the rapid turnover of E3–substrate interactions, their typically low-affinity binding, and the often low abundance of substrates in the cell. However, novel Hakai substrates were discovered involved in other pathways. Hakai interacts with Ajuba through its *lin-11, Isl-1, mec-3* (LIM) domains. Ajuba is a member of the LIM domain-containing protein family (group 3), which has multiple biological implications, including the development of epidermal tissue and oncogenesis [33]. Moreover, it is involved in cell proliferation of skin stem cells through the Hippo/Wnt signaling pathway, in cell cycle progression through Aurora-A, Aurora-B, and CDK1 and in epidermal differentiation through Notch signaling and the stabilization of catenins and actin [33]. Ajuba and Hakai interact through the HYB domain of Hakai and the LIM domain of Ajuba, and both colocalize in the cytoplasm. Hakai promotes Ajuba degradation independent of the E3 ubiquitin ligase activity. Instead, Hakai causes neddylation of Ajuba, which leads to its degradation, requiring the HYB domain for this activity [34]. This dual role of Hakai acting as both, ubiquitin and ubiquitin-like ligase, was also reported to other members of the Cbl family. For example, a dual role as an E3 ubiquitin ligase and as an E3 NEDD8-ligase has been reported for c-Cbl. The overexpression of c-Cbl stabilizes the TGF-β type II receptor TβR-II via neddylation, antagonizing its ubiquitination, which instead targets it for degradation [35]. Functionally, Ajuba depletion has important consequences for malignant transformation. In hepatocellular carcinoma (HCC) cells, Ajuba functions as a tumor suppressor [34], negatively regulating the Wnt signaling pathway. At the molecular level, depletion of Ajuba leads to E-cadherin loss, β-catenin translocation from cell‒cell contacts to the cytoplasm and the nucleus, and increased cyclin D1 and yes-associated protein (YAP) levels [34]. The expression of the YAP target gene CYR61 is increased in Ajuba-depleted HCC cells. On the other hand, overexpression of Ajuba decreases the invasive and migratory capabilities of HCC cells. Although the kinase GSK-3β regulates β-catenin activity through the Wnt/β-catenin pathway, its depletion in Ajuba-depleted HCC does not affect β-catenin translocation or cyclin D1 expression levels [34]. β-catenin translocation is dependent on its interaction with Hakai through the HYB domain, and cells overexpressing Hakai have increased cyclin D1 protein levels. The overexpression of Hakai in HCC results in increased invasion, colony formation and spheroid formation, effects that are reversed by the knockdown of β-catenin. On the other hand, depletion of Hakai decreases the invasion and colony formation ability of HCC. Although Hakai contributes to the degradation of Ajuba and Ajuba is linked to the Wnt pathway, the molecular mechanism and functional role of Hakai in Wnt signaling remains to be further explored.

The HYB domain of Hakai has other interacting proteins described in a Src phosphorylation-dependent manner, including cortactin and docking protein 1 (DOK1). However, although these interactions have been biochemically confirmed the activity-dependent mediation and its functional consequences are still unknown [36]. Both E-cadherin and cortactin are downregulated in stably Hakai-expressing cells [37]. Cortactin is an F-actin binding protein that is phosphorylated by tyrosine kinases (including Src), and its dephosphorylation is a key step in TGFB1-induced EMT [38]. Moreover, downregulation of cortactin enhances the ability of TGFB1 to induce EMT (for example, downregulating E-cadherin) and promote cell migration [38]. On the other hand, DOK1 is an adapter protein that inhibits growth factor and immune regulatory pathways, but it is often downregulated in cancer. Its localization in the cell determines its function, as cytoplasmic DOK1 activates peroxisomes. At present, the interactions described of Hakai with Cortactin and DOK 1 still await further study to determine whether ubiquitination takes place and the biological implications of these interactions.

### Role of Hakai in cellular signaling and regulation mechanisms

#### Implication of Hakai in several signaling pathways

Rack1 is an inhibitor of Src phosphorylation activity and its expression limits Src phosphorylation of E-cadherin, p120-catenin, and β-catenin, localizing them at cell‒cell contacts. As Src phosphorylation is required for Hakai binding and ubiquitination of E-cadherin, Rack1 prevents the lysosomal degradation of E-cadherin [39]. The tyrosine kinase Fyn, a member of the Src protein family, mediates the downregulation of E-cadherin induced by TGFB1. This signaling pathway is dependent on p38 kinase and SNAIL, which is an upregulated E-box transcriptional repressor [40]. In addition, Fyn is involved in the downregulation of E-cadherin by interferon gamma (IFNγ), a proinflammatory cytokine [41]. IFNγ induces the internalization of E-cadherin in a Fyn-dependent manner. The ubiquitination of E-cadherin by Hakai is also increased by IFNγ, leading to its degradation in the proteasome [41]. Given that proinflammatory cytokines, such as interferons (IFNγ), can enhance the immune system’s ability to recognize and kill cancer cells by activating cytotoxic T cells and natural killer (NK) cells, this opens a new important field of investigation regarding the potential role of Hakai in the tumor microenvironment.

On the other hand, the Cdc42 tyrosine kinase contributes to breast cancer invasion and metastasis through the EGFR signaling pathway. Cdc42 inhibits c-Cbl ubiquitination of EGFR, limiting its degradation [42]. The increased protein level of EGFR decreases E-cadherin levels at adherens junctions, trafficking E-cadherin through Src- and Hakai-mediated phosphorylation and ubiquitination for lysosomal degradation, ultimately resulting in EMT [42].

The juxtamembrane domain (JMD) at the cytoplasmic domain of E-cadherin can also bind the protein p120-catenin [43]. This interaction competes with Hakai binding to E-cadherin, whereas the ubiquitination of E-cadherin inhibits its binding to p120-catenin [43]. On the other hand, E-cadherin mutants in the p120-catenin binding domain that are not able to bind p120-catenin have increased interactions with Hakai [44]. Another kinase, spleen tyrosine kinase (SYK), is also involved in the phosphorylation of E-cadherin [45]. The phosphorylation of E-cadherin by SYK also phosphorylates the key tyrosine residues involved in the interaction of Hakai with E-cadherin, but SYK and Hakai do not interact; instead, the phosphorylation of E-cadherin by SYK enhances its interaction at adherens junctions with p120-catenin, also inhibiting cell migration and tumor invasion [45].

#### Regulation of δ-catenin by Hakai

Hakai interacts with Src via a mechanism that regulates the stability of δ-catenin. δ-Catenin is a protein that promotes E-cadherin internalization, releasing β-catenin, which is trafficked to the nucleus, where it mediates oncogenic signaling [46]. δ-Catenin expression levels are correlated with Hakai expression levels in prostate carcinoma cell lines and in kidney cell lines that lack activated Src [47]. However, Hakai and δ-catenin do not physically interact, and they do not colocalize in cells; δ-catenin is associated mainly with the plasma membrane, where Hakai is rarely present [47]. Overexpression of Hakai increases δ-catenin stability. δ-Catenin gain by Hakai overexpression is reversed in cells transfected with Src siRNA, indicating the need for tyrosine phosphorylation of δ-catenin by Src for the stabilizing effect of Hakai on δ-catenin [47]. Nevertheless, this effect is independent of Hakai E3-ubiquitin ligase activity (as confirmed by Hakai mutants that lack the domains needed for this activity). Mechanistically, GSK-3β phosphorylates δ-catenin, resulting in its ubiquitination and ultimately its proteolysis in the proteasome, whereas the phosphorylation of δ-catenin by Src in turn enhances its stability by decreasing its affinity to GSK-3β, through a Hakai-mediated mechanism. In fact, the stabilization of δ-catenin by Hakai is a consequence of the stabilization of Src by Hakai. Hakai overexpression results in an increased stability of Src [47]. The mechanism by which Hakai stabilizes Src remains to be fully elucidated.

#### Regulation of Hakai by Hsp90

Heat shock protein 90 (Hsp90) is a molecular chaperone that plays a crucial role in stabilizing and regulating various client proteins involved in different cellular processes, including cancer [48, 49]. Hakai is regulated by Hsp90 and has been described as a novel client protein for Hsp90. An interaction complex between Hsp90, Hakai, and annexin A2, a calcium-binding protein with roles in membrane and vesicle trafficking, has been reported [49]. Hakai overexpression reduces annexin A2 expression but does not affect Hsp90 expression, whereas silencing Hakai increases annexin A2 expression but has no effect on Hsp90 expression. Inhibition of Hsp90 activity prevents the formation of the complex and decreases Hakai expression while increasing annexin A2 expression [49]. A proposed model for the interaction complex states that Hsp90 stabilizes Hakai, which similarly to its role in E-cadherin degradation, is also responsible for annexin A2 ubiquitination, which leads to its degradation. When Hsp90 is inhibited, Hakai is then degraded through the lysosome, increasing annexin A2 expression [49]. Moreover, geldanamycin-induced Hakai degradation is linked to increased E-cadherin and annexin A2 expression. Additionally, geldanamycin reduces cell motility partly by affecting Hakai expression. These findings identify Hakai as a novel Hsp90 client protein and suggest that Hsp90 inhibitors could be explored for colorectal cancer therapy through their action on Hakai. Later, the alkaloid daurisoline, derived from the plant *Menispermum dauricum*, was shown to target Hsp90, resulting in an increased degradation of Hakai and decreased ubiquitination and degradation of E-cadherin, both in vitro and in vivo, with important implications for tumor growth, EMT markers, and angiogenesis [50].

### Hakai as a component of the nuclear m^6^A methyltransferase complex

Hakai is involved in several other processes independent of its canonical role related to its E3 ubiquitin ligase activity [51]. This was first evidenced by Hakai localization in the nucleus in several cell lines and in cells that do not express E-cadherin, suggesting additional roles independent of its action on E-cadherin [18]. The first reported role for Hakai in the nucleus was its interaction with the nuclear protein polypyrimidine tract-binding protein-associated splicing factor (PSF), an RNA-binding protein [18]. This protein is involved in transcription, DNA binding, unwinding and repair, pre-mRNA splicing, and RNA editing [52]. Both Hakai and PSF colocalize in the nucleus, and their interaction mediates the binding of PSF to its target mRNAs. Hakai overexpression enhances PSF binding to mRNAs encoding cancer-related proteins, whereas Hakai knockdown diminishes the RNA-binding capacity of PSF [18]. Nevertheless, there is no evidence that Hakai is directly responsible for the ubiquitination of PSF [53].

Since then, Hakai has emerged as a component of the m^6^A methyltransferase complex in the nucleus. *N*^6^-methyladenosine (m^6^A) is the most common epigenetic modification of mRNAs in eukaryotes, and it is involved in several cellular processes, including nuclear export, cell cycle regulation, splicing regulation, and stability of mRNAs, among others [54]. In plants and metazoans, it is linked to early pattern formation, whereas in mammals, including humans, it is also associated with several diseases. For example, it is involved in tumor progression and metastasis [55, 56]. m^6^A modification of mRNAs involves complex interactions among writers, readers, and erasers. Writers are the enzymes responsible for adding m^6^A modifications to mRNA transcripts. The main writer complex is known as the m^6^A methyltransferase complex. Readers are proteins that recognize and bind to m^6^A-modified mRNAs, and erasers are enzymes responsible for removing m^6^A modifications from mRNAs [57]. The m^6^A methylosome writer complex [58] consists of an enzymatic core of two methyltransferases or m^6^A METLL complex (MAC), namely, methyltransferase-like protein 3 (METTL3) and methyltransferase-like protein 14 (METTL14) and associated auxiliary proteins or m^6^A–METLL-associated complex (MACOM), including Wilm’s tumor 1-associated protein (WTAP), virilizer-like m^6^A methyltransferase-associated protein (VIRMA/KIA1429), RNA-binding protein 15 (RBM15), and zinc finger CCCH-type containing 13 (ZC3H13), BCLAF1, THRAP3, and Hakai. The MAC is a heterodimer consisting of METLL3, which has methyltransferase activity, and METLL14, which supports the interactions with RNA targets. WTAP stabilizes the interaction of the MAC and recruits the heterodimer into nuclear speckles, where several proteins involved in gene expression and post-transcriptional modifications are found and where the m^6^A modification takes place [54]. WTAP is an essential protein for early embryo development in mice and is involved in cell cycle progression and RNA processing. Hakai interacts with WTAP, and the interaction is dependent on Hakai’s RING finger domain [54].

Hakai was first revealed to be part of the m^6^A writer complex in the plant *Arabidopsis thaliana* and was shown to interact with MTB (the ortholog in plants to METLL14) and to be necessary for mRNA methylation in plants [59]. Mutants of Hakai and other members of the m^6^A writer complex in *A. thaliana* revealed the role in plants of this modification in the response to environmental stress such as the salt stress response [60] and the defence response to pathogens [61], among others. Hakai is also a confirmed member of the complex in the fly *Drosophila melanogaster* [62] on which a stable complex is formed with Fl(2)d (WTAP in humans), virilizer (VIRMA), Flacc (ZC3H13), and Hakai. This complex is thought to act as a platform to connect the other members of the complex and integrate molecular signals to regulate m^6^A modification [62]. Indeed, depletion of Hakai leads to reduced protein levels of the other members of this complex and impairs m^6^A methylation [62]. Hakai interacts with Virilizer through its N-terminal domains, which has also been confirmed in humans (where it interacts with its homolog VIRMA). VIRMA acts as a scaffold protein to allow the interaction of Hakai with WTAP. However, despite dimerization and the RING domain being important for the stabilization of members of the MACOM, Hakai is not reported to possess ubiquitin ligase activity toward them in *Drosophila* [58, 62]. Two zinc finger proteins interact with Hakai in the m^6^A complex of *Arabidopsis*, namely HIZ1 and HIZ2 [63]. HIZ1 expression is regulated by Hakai but not at a post-translational level. In addition, Hakai is necessary for HIZ1 interaction with MTA (METLL3 in humans) but not for HIZ2 interaction. HIZ2 is proposed as the plant equivalent of ZC3H13 [63]. VIRMA has been proposed as a scaffold protein for WTAP/Hakai/ZC3H13 to form a pocket for writers METTL3/METTL14, which is necessary for guiding m^6^A modification in the 3′ untranslated region (UTR) and near stop codons and the 3′UTR of mRNA [64]. Indeed, VIRMA associates with METLL3 and is required for m^6^A writer activity [65]. On the other hand, ZC3H13 is involved in the correct localization of the WTAP/Hakai/VIRMA/ZC3H13 complex in the nucleus, whereas the depletion of ZC3H13 leads to decreased levels of VIRMA, WTAP, and Hakai in the nucleus and the translocation of the complex to the cytoplasm [66]. In humans, VIRMA, Hakai and ZC3H13 are critical for m^6^A methylation, as their silencing in vitro leads to significant decreases in the m^6^A levels of polyadenylated mRNAs [64]. An active chemical compound called ginsenoside Rh2, derived from ginseng (*Panax ginseng*), has been shown to reduce m^6^A methylation levels in several cancer cell lines. Ginsenoside Rh2 has tumor-suppressive activity and downregulates the mRNA levels of Kinesin family member 26B (*KIF26B*), a kinesin motor protein [67]. At the molecular level, KIF26B interacts with ZC3H13 and Hakai in the cytoplasm, increasing the translocation of these proteins to the nucleus. The HYB domain of Hakai is necessary for this interaction [67]. Since m^6^A RNA methylation takes place in the nucleus, KIF26B might enhance m^6^A modification by promoting the nuclear localization of ZC3H13 and Hakai. Although it is clear that Hakai belongs to m^6^A complex, further studies are essential to comprehensively elucidate the role of Hakai in the m^6^A complex, particularly in humans. Although the RING and HYB domains and dimerization are crucial for Hakai’s stabilization of the complex and its “bridging” function among complex members, the exact contribution of the E3 ubiquitin ligase activity of Hakai to its interactions with the m^6^A writer complex and the resulting functional implications for m^6^A regulation remain unclear.

### Implications of Hakai in cellular processes and pathology

#### Hakai role in cell proliferation

So far, Hakai has been implicated primarily in the process of EMT through its action on E-cadherin. However, it is also reported to be involved in cell proliferation via a mechanism independent of E-cadherin expression [18]. Indeed, cells overexpressing Hakai have been found to proliferate at a higher rate than parental cells. This effect was observed even in cells that do not express E-cadherin, such as the HEK293 cell line. The RING-finger domain of Hakai is necessary for this increase in cell proliferation. On the other hand, Hakai-knockdown cells are less proliferative and have lower expression of the cell cycle regulator cyclin D1. In vivo experiments have confirmed that Hakai overexpression enhances tumor formation and proliferation and promotes both invasion and metastasis [19]. Moreover, enhanced Hakai expression is also observed in proliferative tissues such as the endometrium and the lymph nodes, which do not express E-cadherin. The enhanced proliferative effects of Hakai are associated with its oncogenic potential. Hakai also has increased expression in colon adenocarcinoma, gastric adenocarcinoma, and non‐small lung cancer tissues compared with adjacent nontumor tissues [18]. The precise mechanism by which Hakai may influence proliferation is still unclear and whether the HYB domain and the E3 ligase activity are involved remain unknown. However, it was reported that the microRNA (miR)-203 downregulates Hakai expression in epithelial cells [20]. By targeting Hakai with miR-203, an antiproliferative effect is observed in epithelial cells and nonepithelial cells, suggesting that the antiproliferative effect of miR-203 is independent of E-cadherin expression [20]. Moreover, it was shown that Hakai is overexpressed in colon adenocarcinomas, whereas miR-203 is reduced in colon tumors compared with normal colon tissue [20]. Finally, a recent study identified Hakai as an interactor of the transcription factor N-Myc through mass spectrometry analysis in HEK293 embryonic cells [68]. This interaction was further validated by immunoprecipitation. In Wilm’s tumor, the most common type of pediatric renal cancer, a positive correlation between the expression of *MYCN* and *CBLL1* mRNA was found [68]. Downstream genes regulated by N-Myc are involved cell proliferation and control of the cell cycle. The significance of the interaction of Hakai with N-Myc and its possible role in tumor cell proliferation require further research.

#### Hakai role in stemness

Increasing evidence shows the link between EMT and the ubiquitination process in the development and the maintenance of cancer stem cells (CSCs) [69, 70], where E3 ubiquitin ligases play a fundamental role [71]. Other E3 ubiquitin ligases have been reported to be involved in EMT and CSCs. For instance, downregulation of the FBXW7 E3 ligase induces the acquisition of CSC properties and enhances EMT and metastasis in colorectal cancer (CRC) cells [72], NEDD4 is involved in the maintenance of CSC properties in breast cancer [73], and the pharmacological inactivation of Skp2 could reduce the self-renewal capability of CSCs [74]. Given the involvement of Hakai in EMT, the possible role of Hakai in the acquisition of stem properties has gained importance. In a tumorsphere in vitro model, the silencing of Hakai with a specific shRNA-*CBLL1* resulted in reduced tumorsphere number and sizes, together with the downregulation of Lgr5, probably the best established colon cancer stem cell marker, and Nanog and Klf4, universal CSC markers, at the protein level [75]. Despite these efforts, the specific mechanism by which Hakai is involved in cancer stem cells still remains unknown. On the other hand, the importance of Hakai functions in embryonic development has also been reported in *D. melanogaster* [76]. The Hakai homolog in *D. melanogaster* conserves the RING domain and interacts with the E-cadherin homolog, which is crucial for embryonic development [76]. The role of Hakai in m^6^A methylation has also been linked to the embryonic development in *A. thaliana* studies [77]. Indeed, knockdown of Hakai, similar to the knockdown of WTAP, virilizer, and ZC3H13 impairs self-renewal and triggers differentiation in mouse embryonic stem cells [66], further supporting Hakai role in stemness. Other developmental studies in vivo models showed that Hakai is essential in the early developmental stages of the *D. melanogaster* life cycle (embryogenesis) [76]. In *Drosophila*, Hakai, and E-cadherin form a complex differently than they do in mammals. Hakai null mutants died at the larval stage, but this phenomenon was reversed by the HA-tagged Hakai construct. While zygotic Hakai was not required for cell proliferation and differentiation in the wing disc epithelium, maternal Hakai mutants presented defects in epithelial integrity, including stochastic E-cadherin loss, reduced αKC levels, and issues related to cell specification and migration. However, E-cadherin levels did not increase. Thus, Hakai may regulate other proteins essential for early embryonic morphogenesis in *Drosophila*. The Hakai homolog in *Drosophila* is expressed in the cytoplasm of cells, which points to an indirect interaction through another molecule, unlike what happens in mammalian Hakai and E-cadherin, which directly interact [76]. Moreover, Hakai overexpression in *D. melanogaster* did not decrease E-cadherin levels at cell‒cell contacts, suggesting a different role for this interaction [76]. Thus, *Drosophila* Hakai may have different interacting partners that mediate cell adhesion and migration, which are essential for early embryonic morphogenesis [76]. Finally, an important study has revealed that E-cadherin ubiquitination by Hakai might play a major role in human embryonic stem cells [78]. Consistent with its role, Hakai knockdown increased E-cadherin and β-catenin levels, enhancing morphogen-stimulated mesoderm differentiation in human embryonic stem cells cultured on stiff gels. These findings suggest that on a stiff substrate, increased Hakai activity promotes E-cadherin internalization, destabilizes adherens junctions and releases β-catenin into the cytoplasm, where it is quickly degraded in the proteasome, thus reducing its ability to induce mesoderm differentiation [78]. Therefore, an increasing body of evidence is beginning to highlight the potential role of Hakai in stemness and cancer stem cells; however, further studies are essential to determine its impact and implications in human cancer stemness.

#### Implications of Hakai in cancer

Numerous bioinformatics studies have highlighted the potential diagnostic and prognostic value of m^6^A regulators in various diseases [79–85], including lung, hepatocellular, esophageal, ovarian, prostate, cervical, and breast cancers (summarized in Table 1). Despite extensive evidence implicating Hakai in the m^6^A writer complex—including functional studies across various models and numerous gene signatures of m^6^A-related genes in different diseases—the specific role of Hakai in m^6^A modification remains to be fully explored. Here, we present evidence of Hakai’s involvement in various cancer types, informed by bioinformatics analyses that, in many cases, require further experimental validation.

**Table 1:** Bioinformatic analysis of m^6^A-related genes and their predictive value for patient prognosis

Disease	Genes	Purpose	*CBLL1* expression	Database	Reference
Hepatocellular carcinoma	*METTL3, WTAP, RBM15, RBM15B, VIRMA, CBLL1, METTL14, ZC3H13*	Prognosis and tumor immune infiltration	Upregulated	TCGA and GEO	[101]
Lung cancer	*ZC3H13, CBLL1, ELAVL1, YTHDF1*	Prognosis, tumor immune infiltration and drug response	Downregulated	TCGA and GEO	[102]
Lung adenocarcinoma	*METTL3, KIAA1429, HNRNPC, YTHDF1, YTHDF2, IGF2BP1, IGF2BP2, IGFBP3, FMR1, LRPPRC, HNRNPA2B1*	Prognosis, molecular subtype clustering	Not differentially expressed and correlates to LAG3 (lymphocyte activated gene 3) that is involved in Treg suppressive function	TCGA	[85]
Lung adenocarcinoma	*HNRNPA2B1, HNRNPC, IGF2BP2, IGF2BP3, LRPPRC, RMB15, WTAP, ZC3H13*	Prognosis, molecular subtype clustering	Not differentially expressed and differentially expressed in different tumor molecular clusters	GEO, TCGA	[103]
Early-stage lung adenocarcinoma	*LRIG1, CTSV, KIF20A, ATP13A3, TMPRSS2*	Prognosis, molecular subtype clustering, immune infiltration	Not differentially expressed and upregulated in CD4^+^ T cells and regulatory T cells	TCGA, GEO, single-cell transcriptome database [104]	[105]
Esophageal cancer	*HNRNPC, YTHDC2, WTAP, VIRMA, IGF2BP3, HNRNPA2B1*	Prognosis, immune infiltration	Upregulated	TCGA	[106]
Cervical Cancer	*WTAP, RBM15, CBLL1, YTHDC2*	Diagnostic	Downregulated	GEO and m6a2target	[107]
Prostate cancer	*HNRNPA2B1, CBLL1, FTO, YTHDC1, HNRNP, WTAP*	Methylation prognosis model	Downregulated	TCGA	[108]
Breast cancer	*CBLL1*	Prognostic	Upregulated Clustering based on high or low expression	TCGA	[96]
Ovarian cancer	*KIAA1429, WTAP, SNAI1, AXL, IGF2BP1, ELAVL1, CBLL1, CDH2, NANOG, ALKBH5*	Prognostic	Upregulated	TCGA, GTEx	[109]
Ovarian cancer	*CBLL1, FTO, HNRNPC, METTL3, METTL14, WTAP, ZC3H13, RBM15B, YTHDC2*	Prognostic	Upregulated	TCGA, GTEx	[110]

##### Colorectal and gastric cancer

The role of Hakai in cancer was first reported in gastric and CRC tissues, where Hakai is highly expressed compared with adjacent nontransformed epithelial tissues. Hakai expression gradually increases in colon carcinoma from stage I to stage IV, suggesting its potential use as a biomarker of tumor progression [19]. Later, the potential of Hakai for the stratification of patients with CRC was studied on the basis of the most widely used molecular classification of CRC: the consensus molecular subtype (CMS) classification [86]. This classification system is based on transcriptomics analysis, integrates phenotype and clinical characteristics, and is considered the best approach to date for cancer molecular classification. This system may be used for future clinical stratification and help in the design of targeted interventions. In the CMS system, CRC can be classified as CMS1 (immune subtype, with microsatellite instability and strong immune activation), CMS2 (canonical subtype, characterized by epithelial and WNT, MYC and EGFR signaling activation), CMS3 (metabolic subtype, epithelial with metabolic dysregulation), or CMS4 (mesenchymal subtype, with activation of TGFB1, stromal invasion and angiogenesis). High *CBLL1* gene (Hakai protein) expression is specifically associated with CMS2 in CRC (the canonical subtype), which is characterized by the activation of WNT, MYC, and EGFR signaling and high expression of cyclins [75]. Moreover, high expression of Hakai in CMS2 patients was correlated with worse overall survival [75]. Thus, Hakai is posed as a novel biomarker of CMS2 CRC, with the potential to stratify patients with poor overall survival.

Recent studies have emphasized the importance of the Slit2-Robo1 signaling in migration, invasion, and tumor metastasis. Both Slit2 and Robo1 are overexpressed in CRC [87] and their expression is associated with an increased risk of metastasis and poorer overall survival in patients [87]. Slit2 is secreted by solid tumors and binds to plasma-membrane-bound Robo1 expressed by colorectal epithelial carcinoma cells. Slit-Robo signaling recruits Hakai to E-cadherin, causing its ubiquitination and lysosomal degradation, thus playing a role in the malignant transformation of these tumors [87]. On the other hand, in hereditary diffuse gastric cancer, missense mutations in E-cadherin are relatively frequent (~30%) and result in decreased binding to p120-catenin but increased binding to Hakai, resulting in increased invasiveness [44].

Moreover, Hakai expression has also been studied in inflammatory bowel disease (IBD), which increases the risk of colorectal cancer (CRC) and includes conditions such as ulcerative colitis (UC) and Crohn’s disease (CD). Hakai expression is upregulated in UC and CD biopsies compared with normal tissues, and higher expression was even detected in TNM stage IV of CRC tissues. However, these results were not replicated in IBD mouse models, suggesting that Hakai regulation in mice does not accurately mimic human IBD [88]. Although mouse models have been widely used to study basic pathophysiological mechanisms, significant controversies exist regarding how well these models reflect human inflammatory diseases.

##### Liver cancer

The role of Hakai in hepatocellular carcinoma is particularly relevant because of its action on the Ajuba protein, which mediates tumor cell proliferation [34, 89]. As previously mentioned, Ajuba degradation by Hakai has important consequences for malignant transformation. Although, so far, little is known regarding the role of Hakai in the tumor microenvironment [90], acidic pH growth medium results in the phosphorylation of p120-catenin, resulting in its dissociation from E-cadherin, coupled with the phosphorylation of E-cadherin by Src in hepatoblastoma cells. This, in turn, allows the ubiquitination of E-cadherin by Hakai as well as the degradation, increasing the migratory and invasive capabilities of these cells [90]. Further studies to elucidate the role of Hakai in the tumor microenvironment are needed.

##### Non‑small cell lung cancer

Another interesting study was focused on the role of Hakai in regulating cell growth, invasion, and chemosensitivity to cisplatin in non‑small cell lung cancer (NSCLC) [91]. The downregulation of Hakai causes the upregulation of E-cadherin and the downregulation of N-cadherin expression, limiting cancer cell migration and invasion capabilities [91]. Moreover, it also decreases the phosphorylation of AKT (Ser473), which is normally hyperactivated in NSCLC, potentially explaining its ability to inhibit growth through AKT signaling [91]. A link between AKT phosphorylation and resistance to cisplatin has been previously reported, and Hakai silencing can, therefore, sensitize NSCLC to cisplatin [91]. Hakai is overexpressed in NSCLC tissue compared with adjacent nontumor tissue, and its levels are correlated with tumor size [21]. At the molecular level, both cell cycle regulating proteins cyclin D1 and cyclin-dependent kinase 4 (CDK4) are downregulated in Hakai-knockdown cells, increasing the percentage of cells in the G1 phase of cell cycle arrest. Hakai knockdown also increases E-cadherin protein levels while decreasing the expression of matrix metalloproteinases MMP2 and MMP9, which are key factors in lung cancer invasion and metastasis [21]. Importantly, it is well known that EGFR mutations are relatively common in lung cancer, and even though EGFR-tyrosine kinase inhibitors (TKIs) are effective for patients, the development of resistance is a major cause of failure of this treatment [92]. Lung cancer cells with acquired resistance have downregulated E-cadherin expression and EMT characteristics. Decreased levels of E-cadherin are correlated with decreased expression of programmed death ligand 1 (PD-L1) [92]. This finding has important implications for these patients, as immune checkpoint inhibitors are proposed for treatment when tumors acquire resistance to EGFR-TKIs. Thus, downregulation of E-cadherin and acquisition of EMT characteristics in EGFR-TKI-resistant lung cancer cells, which correlate with reduced expression of PD-L1, might limit the effectiveness of this treatment strategy [92]. Interestingly, EFGR-TKI gefitinib-resistant cells exhibit Src activation and Hakai upregulation [93]. Knockdown of Hakai results in increased E-cadherin expression, reduced stemness, and resensitization to gefitinib [93]. Treatment of resistant cells with the dual inhibitor JMF3086, which simultaneously targets HMGR and HDAC, results in decreased *Hakai* transcription and Src inactivation, which in turn increases E-cadherin protein levels and reduces vimentin expression and stemness while restoring EGFR-TKI sensitivity [93]. Taken all together, targeting Hakai in chemotherapy resistant non‑small cell lung cancer would be an interesting strategy to explore.

##### Breast cancer

Approximately one-third of breast cancers lack estrogen receptor α (ERα), which is important for the response to estrogen and regulates proliferation and tissue development. ERα^−^ cancers have a poor prognosis, do not respond to hormone response modifiers, and are often resistant to chemotherapy [94]. Estrogen regulates the transcriptional activation and ubiquitin-dependent proteolysis of ERα in cooperation with Src [94]. The Src protein kinase phosphorylates ERα and enhances its affinity for estrogen. In primary breast cancer, the levels of Src and ERα are inversely correlated. Both proteasome and Src inhibitors increased ERα levels in cell lines. The inhibition of Src also impairs ligand-activated ERα ubiquitination. Src siRNA reversed the ligand-activated ERα loss [94]. In breast cancer cells, Hakai bind to ERα through its DNA-binding domain, resulting in the inhibition of its transcriptional activity, thus regulating the expression of ERα target genes [95]. This inhibition was reported to be independent of Hakai ubiquitin ligase activity, as it is based on Hakai competition with coactivators of ERα, including Src. estrogen-dependent proliferation and migration in breast cancer cells are inhibited by Hakai [95], in contrast with the proliferative estrogen-independent effects reported in other cell lines [18]. Conversely, the prognostic value of *CBLL1* gene expression in breast carcinomas was analyzed alongside that of other m^6^A regulators. High *CBLL1* gene expression was associated with a better prognosis in patients with breast cancer, and functional analysis revealed its involvement in the regulation of multiple pathways, including apoptosis, the ESR1-pathway, and immune response. On the other hand, low expression of *CBLL1* is associated with tamoxifen resistance [96]. These results suggest that a noncanonical mechanism can be implicated in breast cancer, where the ubiquitin ligase activity is not involved, further suggesting that the E3 ligase activity of Hakai may not be involved in hormone-dependent breast cancer. It would be important to reproduce these results and further investigate whether other hormone-dependent cancers have similar behavior.

#### E3 ubiquitin ligase activity of Hakai as a promising therapeutic strategy against cancer

Previous reports highlight the role of Hakai in tumor progression and metastasis, making it a promising drug target for cancer treatment. As previously mentioned, the novel HYB domain, structurally different from other PTB domains, represents a highly suitable target for therapeutic intervention. In 2020, a novel class of specific inhibitors targeting the HYB domain of Hakai was identified via virtual screening. A novel small-molecule inhibitor, called Hakin-1, was designed to disrupt the phosphorylated-E-cadherin binding site of Hakai [97]. By effectively blocking the Hakai-mediated ubiquitination of E-cadherin, Hakin-1 prevents its degradation. In preclinical studies using tumor xenograft models in mice, Hakin-1 demonstrated significant efficacy in inhibiting tumor growth and lung metastasis without observable toxicity effects [97]. While Hakin-1 represents the first reported inhibitor specifically targeting the HYB domain of Hakai, several nutraceuticals, such as celastrol, vinflunine, or silibinin, have also been documented to influence Hakai in several types of cancers [98–100]; however, none of these nutraceuticals are reported to directly and specifically target Hakai.

## Conclusions

In this review, we highlight the latest knowledge available regarding the protein Hakai and its canonical and noncanonical emerging functions. Hakai is an important regulator of adherens junctions and a posttranslational regulator of E-cadherin at cell‒cell contacts, which leads to epithelial cell plasticity and EMT. As novel interactions of this protein have been revealed, the number of cellular processes in which we now know Hakai is involved has increased, highlighting its importance as a drug target for targeted therapies. Hakai was reported to be a regulator of the stability of other Src kinase substrates, and further studies are needed to fully understand its impact on different signaling pathways. More studies are needed to better understand the role of Hakai in proliferation and in cancer cell stemness. The role of Hakai in the nucleus has been revealed, as has its involvement in the m^6^A writer complex. The mechanisms of the interaction of Hakai with the m^6^A writer complex are still not fully understood but have tremendous biological consequences. Finally, the potential of Hakai as a biomarker and its prognostic value in cancer also reveals its untapped potential in precision medicine. The development of the first allosteric inhibitor that targets its atypical substrate binding-domain is a promising step that could eventually lead to patient benefit.

## Data Availability

Not applicable.

## Abbreviations

ALKBH5: Alpha-ketoglutarate-dependent dioxygenase AlkB homolog 5
CAMSAP3: Calmodulin-regulated spectrin-associated protein 3
Cbl: Casitas B-lineage lymphoma
CBLL1: Cbl proto-oncogene-like 1
CD: Crohn’s disease
CDK4: Cyclin-dependent kinase 4
CIRC_0072083: Circular RNA 0072083
CMS: Concensus molecular subtype
CRC: Colorectal cancer
CSCs: Cancer stem cells
DOK1: Docking protein 1
DUBs: Deubiquitinases
EGFR: Epithelial growth factor receptor
ELAVL1: Embryonic lethal abnormal vision-like protein 1
EMT: Epithelial-to-mesenchymal transition
ERα: Estrogen receptor α
FMR1: Fragile X messenger ribonucleoprotein 1
FTO: Fat mass and obesity-associated protein
4H: Four-helix bundle
HCC: Hepatocellular carcinoma
HECT: Homologous to E6-associated protein carboxyl terminus
Hsp90: Heat shock protein 90
HYB: Hakai-phosphotyrosine-binding
IBD: Inflammatory bowel disease
IBR: In-between RING
IGF2BP1: Insulin-like growth factor-2 mRNA binding protein 1
IGF2BP2: Insulin-like growth factor-2 mRNA binding protein 2
IGF2BP3: Insulin-like growth factor 2 mRNA binding protein 3
IFNγ: Interferon gamma
JMD: Juxtamembrane domain
KIFC3: Kinesin family member C3
LIM: Lin-11, Isl-1, mec-3 domain
m^6^A: *N*^6^-methyladenosine
MAC: M6A METLL complex
MACOM: M6A-METLL-associated complex
METTL3: Methyltransferase-like protein 3
METTL14: Methyltransferase-like protein 14
miR: MicroRNA
NSCLC: Non‑small cell lung cancer
PTB: Phosphotyrosine binding domain
PD-L1: Programmed death ligand 1
PLEKHA7: Pleckstin homology domain-containing family A member 7
PSF: Polypyrimidine tract-binding protein-associated splicing factor
pTYR: Phospho-tyrosine
RBR: RING-between-RING
RING: Really interesting new gene
SH2: Src homology domain
SYK: Spleen tyrosine kinase
TGFB1: Transforming growth factor beta 1
TKB: Tyrosine kinase binding
TKIs: Tyrosine kinase inhibitors
UAB: Ubiquitin associated domain
UC: Ulcerative colitis
USP47: Ubiquitin-specific protease 47
VIRMA: Virilizer-like m^6^A methyltransferase-associated
WTAP: Wilm’s tumor 1-associated protein
YAP: Yes-associated protein
YTHDC: YT521-B homology domain-containing protein
ZC3H13: Zinc finger CCCH-type containing 13

## References

[1] Fujita Y, Krause G, Scheffner M, Zechner D, Leddy HEM, Behrens J, Sommer T, Birchmeier W. Hakai, a c-Cbl-like protein, ubiquitinates and induces endocytosis of the E-cadherin complex. Nat Cell Biol. 2002;4:222–31. 10.1038/ncb758. 11836526 10.1038/ncb758

[2] Hershko A, Ciechanover A. The ubiquitin system. Annu Rev Biochem. 1998;67:425–79. 10.1146/annurev.biochem.67.1.425. 9759494 10.1146/annurev.biochem.67.1.425

[3] Rape M. Ubiquitylation at the crossroads of development and disease. Nat Rev Mol Cell Biol. 2018;19:59–70. 10.1038/nrm.2017.83. 28928488 10.1038/nrm.2017.83

[4] Heideker J, Wertz IE. DUBs, the regulation of cell identity and disease. Biochemical Journal. 2015;465:1–26. 10.1042/BJ20140496. 25631680 10.1042/BJ20140496

[5] Schulman BA, Wade HJ. Ubiquitin-like protein activation by E1 enzymes: the apex for downstream signalling pathways. Nat Rev Mol Cell Biol. 2009;10:319–31. 10.1038/nrm2673. 19352404 10.1038/nrm2673 PMC2712597

[6] Stewart MD, Ritterhoff T, Klevit RE, Brzovic PS. E2 enzymes: more than just middle men. Cell Res. 2016;26:423–40. 10.1038/cr.2016.35. 27002219 10.1038/cr.2016.35 PMC4822130

[7] Pickart CM. Mechanisms underlying ubiquitination. Annu Rev Biochem. 2001;70:503–33. 10.1146/annurev.biochem.70.1.503. 11395416 10.1146/annurev.biochem.70.1.503

[8] Hatakeyama S, Nakayama KI. U-box proteins as a new family of ubiquitin ligases. Biochem Biophys Res Commun. 2003;302:635–45. 10.1016/S0006-291X(03)00245-6. 12646216 10.1016/s0006-291x(03)00245-6

[9] Smit JJ, Sixma TK. RBR E3-ligases at work. EMBO Rep. 2014;15:142–54. 10.1002/embr.201338166. 24469331 10.1002/embr.201338166 PMC3989860

[10] Deshaies RJ, Joazeiro CAP. RING domain E3 ubiquitin ligases. Annu Rev Biochem. 2009;78:399–434. 10.1146/annurev.biochem.78.101807.093809. 19489725 10.1146/annurev.biochem.78.101807.093809

[11] Buetow L, Huang DT. Structural insights into the catalysis and regulation of E3 ubiquitin ligases. Nat Rev Mol Cell Biol. 2016;17:626–42. 10.1038/nrm.2016.91. 27485899 10.1038/nrm.2016.91 PMC6211636

[12] Tang R, Langdon WY, Zhang J. Negative regulation of receptor tyrosine kinases by ubiquitination: key roles of the Cbl family of E3 ubiquitin ligases. Front Endocrinol (Lausanne). 2022. 10.3389/fendo.2022.971162. 35966060 10.3389/fendo.2022.971162 PMC9365936

[13] Mukherjee M, Chow SY, Yusoff P, Seetharaman J, Ng C, Sinniah S, Koh XW, Asgar NFM, Li D, Yim D, et al. Structure of a novel phosphotyrosine-binding domain in Hakai that targets E-cadherin. EMBO J. 2012;31:1308–19. 10.1038/emboj.2011.496. 22252131 10.1038/emboj.2011.496 PMC3298002

[14] Mukherjee M, Jing-Song F, Ramachandran S, Guy GR, Sivaraman J. Dimeric switch of Hakai-truncated monomers during substrate recognition. J Biol Chem. 2014;289:25611–23. 10.1074/jbc.M114.592840. 25074933 10.1074/jbc.M114.592840 PMC4162166

[15] Liu Y-Q, Bai G, Zhang H, Su D, Tao D-C, Yang Y, Ma Y-X, Zhang S-Z. Human RING finger protein ZNF645 is a novel testis-specific E3 ubiquitin ligase. Asian J Androl. 2010;12:658–66. 10.1038/aja.2010.54. 20657603 10.1038/aja.2010.54 PMC3739317

[16] Vleminckx K, Vakaet L, Mareel M, Fiers W, Van Roy F. Genetic manipulation of E-cadherin expression by epithelial tumor cells reveals an invasion suppressor role. Cell. 1991;66:107–19. 10.1016/0092-8674(91)90143-M. 2070412 10.1016/0092-8674(91)90143-m

[17] Christofori G, Semb H. The role of the cell-adhesion molecule E-cadherin as a tumour-suppressor gene. Trends Biochem Sci. 1999;24:73–6. 10.1016/S0968-0004(98)01343-7. 10098402 10.1016/s0968-0004(98)01343-7

[18] Figueroa A, Kotani H, Toda Y, Mazan-Mamczarz K, Mueller E-C, Otto A, Disch L, Norman M, Ramdasi RM, Keshtgar M, et al. Novel roles of Hakai in cell proliferation and oncogenesis. Mol Biol Cell. 2009;20:3533–42. 10.1091/mbc.e08-08-0845. 19535458 10.1091/mbc.E08-08-0845 PMC2719571

[19] Castosa R, Martinez-Iglesias O, Roca-Lema D, Casas-Pais A, Díaz-Díaz A, Iglesias P, Santamarina I, Graña B, Calvo L, Valladares-Ayerbes M, et al. Hakai overexpression effectively induces tumour progression and metastasis in vivo. Sci Rep. 2018;8:3466. 10.1038/s41598-018-21808-w. 29472634 10.1038/s41598-018-21808-w PMC5823865

[20] Abella V, Valladares M, Rodriguez T, Haz M, Blanco M, Tarrío N, Iglesias P, Aparicio LA, Figueroa A. miR-203 regulates cell proliferation through its influence on Hakai expression. PLoS ONE. 2012;7: e52568. 10.1371/journal.pone.0052568. 23285092 10.1371/journal.pone.0052568 PMC3527564

[21] Hui L, Zhang S, Wudu M, Ren H, Xu Y, Zhang Q, Qiu X. CBLL1 is highly expressed in non-small cell lung cancer and promotes cell proliferation and invasion. Thorac Cancer. 2019;10:1479–88. 10.1111/1759-7714.13097. 31124298 10.1111/1759-7714.13097 PMC6558451

[22] Pece S, Gutkind JS. E-cadherin and Hakai: signalling, remodeling or destruction? Nat Cell Biol. 2002;4:E72–4. 10.1038/ncb0402-e72. 11944035 10.1038/ncb0402-e72

[23] Palacios F, Tushir JS, Fujita Y, D’Souza-Schorey C. Lysosomal targeting of E-Cadherin: a unique mechanism for the down-regulation of cell-cell adhesion during epithelial to mesenchymal transitions. Mol Cell Biol. 2005;25:389–402. 10.1128/MCB.25.1.389-402.2005. 15601859 10.1128/MCB.25.1.389-402.2005 PMC538771

[24] Mosesson Y, Mills GB, Yarden Y. Derailed endocytosis: an emerging feature of cancer. Nat Rev Cancer. 2008;8:835–50. 10.1038/nrc2521. 18948996 10.1038/nrc2521

[25] Loh C-Y, Chai J, Tang T, Wong W, Sethi G, Shanmugam M, Chong P, Looi C. The E-cadherin and N-cadherin switch in epithelial-to-mesenchymal transition: signaling, therapeutic implications, and challenges. Cells. 2019;8:1118. 10.3390/cells8101118. 31547193 10.3390/cells8101118 PMC6830116

[26] Rubtsova SN, Zhitnyak IY, Gloushankova NA. Dual role of E-cadherin in cancer cells. Tissue Barriers. 2022. 10.1080/21688370.2021.2005420. 34821540 10.1080/21688370.2021.2005420 PMC9621038

[27] Puisieux A, Brabletz T, Caramel J. Oncogenic roles of EMT-inducing transcription factors. Nat Cell Biol. 2014;16:488–94. 10.1038/ncb2976. 24875735 10.1038/ncb2976

[28] Janda E, Nevolo M, Lehmann K, Downward J, Beug H, Grieco M. Raf plus TGFβ-dependent EMT is initiated by endocytosis and lysosomal degradation of E-cadherin. Oncogene. 2006;25:7117–30. 10.1038/sj.onc.1209701. 16751808 10.1038/sj.onc.1209701

[29] Wang Z, Sandiford S, Wu C, Li SSC. Numb regulates cell–cell adhesion and polarity in response to tyrosine kinase signalling. EMBO J. 2009;28:2360–73. 10.1038/emboj.2009.190. 19609305 10.1038/emboj.2009.190 PMC2712596

[30] Nathan JA, Tae Kim H, Ting L, Gygi SP, Goldberg AL. Why do cellular proteins linked to K63-polyubiquitin chains not associate with proteasomes? EMBO J. 2013;32:552–65. 10.1038/emboj.2012.354. 23314748 10.1038/emboj.2012.354 PMC3579138

[31] Yang Q, Zhao J, Chen D, Wang Y. E3 ubiquitin ligases: styles, structures and functions. Molecular Biomedicine. 2021;2:23. 10.1186/s43556-021-00043-2. 35006464 10.1186/s43556-021-00043-2 PMC8607428

[32] Sako-Kubota K, Tanaka N, Nagae S, Meng W, Takeichi M. Minus end–directed motor KIFC3 suppresses E-cadherin degradation by recruiting USP47 to adherens junctions. Mol Biol Cell. 2014;25:3851–60. 10.1091/mbc.e14-07-1245. 25253721 10.1091/mbc.E14-07-1245 PMC4244195

[33] Schleicher K, Schramek D. AJUBA: a regulator of epidermal homeostasis and cancer. Exp Dermatol. 2021;30:546–59. 10.1111/exd.14272. 33372298 10.1111/exd.14272

[34] Liu M, Jiang K, Lin G, Liu P, Yan Y, Ye T, Yao G, Barr MP, Liang D, Wang Y, et al. Ajuba inhibits hepatocellular carcinoma cell growth via targeting of β-catenin and YAP signaling and is regulated by E3 ligase Hakai through neddylation. J Exp Clin Cancer Res. 2018;37:165. 10.1186/s13046-018-0806-3. 30041665 10.1186/s13046-018-0806-3 PMC6057013

[35] Zuo W, Huang F, Chiang YJ, Li M, Du J, Ding Y, Zhang T, Lee HW, Jeong LS, Chen Y, et al. c-Cbl-mediated neddylation antagonizes ubiquitination and degradation of the TGF-β type II receptor. Mol Cell. 2013;49:499–510. 10.1016/j.molcel.2012.12.002. 23290524 10.1016/j.molcel.2012.12.002

[36] Kaneko T, Joshi R, Feller SM, Li SS. Phosphotyrosine recognition domains: the typical, the atypical and the versatile. Cell Commun Signal. 2012;10:32. 10.1186/1478-811X-10-32. 23134684 10.1186/1478-811X-10-32 PMC3507883

[37] Díaz-Díaz A, Casas-Pais A, Calamia V, Castosa R, Martinez-Iglesias O, Roca-Lema D, Santamarina I, Valladares-Ayerbes M, Calvo L, Chantada V, et al. Proteomic analysis of the e3 ubiquitin ligase hakai highlights a role in plasticity of the cytoskeleton dynamics and in the proteasome system. J Proteome Res. 2017;16:2773–88. 10.1021/acs.jproteome.7b00046. 28675930 10.1021/acs.jproteome.7b00046

[38] Zhang K, Wang D, Song J. Cortactin is involved in transforming growth factor-β1-induced epithelial-mesenchymal transition in AML-12 cells. Acta Biochim Biophys Sin (Shanghai). 2009;41:839–45. 10.1093/abbs/gmp070. 19779649 10.1093/abbs/gmp070

[39] Swaminathan G, Cartwright CA. Rack1 promotes epithelial cell–cell adhesion by regulating E-cadherin endocytosis. Oncogene. 2012;31:376–89. 10.1038/onc.2011.242. 21685945 10.1038/onc.2011.242

[40] Kim AN, Jeon WK, Lim KH, Lee HY, Kim WJ, Kim BC. Fyn mediates transforming growth factor-beta1-induced down-regulation of E-cadherin in human A549 lung cancer cells. Biochem Biophys Res Commun. 2011;407:181–4. 10.1016/j.bbrc.2011.02.134. 21371426 10.1016/j.bbrc.2011.02.134

[41] Smyth D, Leung G, Fernando M, McKay DM. Reduced surface expression of epithelial e-cadherin evoked by interferon-gamma is fyn kinase-dependent. PLoS ONE. 2012;7: e38441. 10.1371/journal.pone.0038441. 22715382 10.1371/journal.pone.0038441 PMC3371038

[42] Shen Y, Hirsch DS, Sasiela CA, Wu WJ. Cdc42 regulates E-cadherin ubiquitination and degradation through an epidermal growth factor receptor to Src-mediated pathway. J Biol Chem. 2008;283:5127–37. 10.1074/jbc.M703300200. 18057010 10.1074/jbc.M703300200

[43] Hartsock A, Nelson WJ. Competitive regulation of E-cadherin juxtamembrane domain degradation by p120-catenin binding and Hakai-mediated ubiquitination. PLoS ONE. 2012;7: e37476. 10.1371/journal.pone.0037476. 22693575 10.1371/journal.pone.0037476 PMC3365061

[44] Figueiredo J, Söderberg O, Simões-Correia J, Grannas K, Suriano G, Seruca R. The importance of E-cadherin binding partners to evaluate the pathogenicity of E-cadherin missense mutations associated to HDGC. Eur J Hum Genet. 2013;21:301–9. 10.1038/ejhg.2012.159. 22850631 10.1038/ejhg.2012.159 PMC3573198

[45] Kassouf T, Larive R, Morel A, Urbach S, Bettache N, Marcial Medina M, Mèrezègue F, Freiss G, Peter M, Boissière-Michot F, et al. The syk kinase promotes mammary epithelial integrity and inhibits breast cancer invasion by stabilizing the E-cadherin/catenin complex. Cancers (Basel). 2019;11:1974. 10.3390/cancers11121974. 31817924 10.3390/cancers11121974 PMC6966528

[46] Kim H, He Y, Yang I, Zeng Y, Kim Y, Seo YW, Murnane MJ, Jung C, Lee JH, Min JJ, et al. δ-Catenin promotes E-cadherin processing and activates β-catenin-mediated signaling: implications on human prostate cancer progression. Biochim Biophys Acta Mol Basis Dis. 2012;1822:509–21. 10.1016/j.bbadis.2011.12.015. 10.1016/j.bbadis.2011.12.015 PMC3763829 22261283

[47] Shrestha H, Ryu T, Seo Y-W, Park S-Y, He Y, Dai W, Park E, Simkhada S, Kim H, Lee K, et al. Hakai, an E3-ligase for E-cadherin, stabilizes δ-catenin through Src kinase. Cell Signal. 2017;31:135–45. 10.1016/j.cellsig.2017.01.009. 28069439 10.1016/j.cellsig.2017.01.009

[48] Somu P, Mohanty S, Basavegowda N, Yadav AK, Paul S, Baek K-H. The interplay between heat shock proteins and cancer pathogenesis: a novel strategy for cancer therapeutics. Cancers (Basel). 2024;16:638. 10.3390/cancers16030638. 38339390 10.3390/cancers16030638 PMC10854888

[49] Díaz-Díaz A, Roca-Lema D, Casas-Pais A, Romay G, Colombo G, Concha Á, Graña B, Figueroa A. Heat shock protein 90 chaperone regulates the e3 ubiquitin ligase Hakai protein stability. Cancers (Basel). 2020;12:215. 10.3390/cancers12010215. 31952268 10.3390/cancers12010215 PMC7017148

[50] Huang K, Chen Q, Deng L, Zou Q, Min S. Daurisoline inhibiting tumor angiogenesis and epithelial-mesenchymal transition in bladder cancer by mediating HAKAI protein stability. Iran J Pharm Res. 2022. 10.5812/ijpr-129798. 36937208 10.5812/ijpr-129798 PMC10016139

[51] Aparicio LA, Valladares M, Blanco M, Alonso G, Figueroa A. Biological influence of Hakai in cancer: a 10-year review. Cancer Metastasis Rev. 2012;31:375–86. 10.1007/s10555-012-9348-x. 22349934 10.1007/s10555-012-9348-x PMC3350634

[52] Figueroa A, Fujita Y, Gorospe M. Hacking RNA: Hakai promotes tumorigenesis by enhancing the RNA-binding function of PSF. Cell Cycle. 2009;8:3648–51. 10.4161/cc.8.22.9909. 19855157 10.4161/cc.8.22.9909 PMC2808762

[53] Dong L, Nian H, Shao Y, Zhang Y, Li Q, Yi Y, Tian F, Li W, Zhang H, Zhang X, et al. PTB-associated splicing factor inhibits IGF-1-induced VEGF upregulation in a mouse model of oxygen-induced retinopathy. Cell Tissue Res. 2015;360:233–43. 10.1007/s00441-014-2104-5. 25638408 10.1007/s00441-014-2104-5

[54] Horiuchi K, Kawamura T, Iwanari H, Ohashi R, Naito M, Kodama T, Hamakubo T. Identification of Wilms’ Tumor 1-associating protein complex and its role in alternative splicing and the cell cycle. J Biol Chem. 2013;288:33292–302. 10.1074/jbc.M113.500397. 24100041 10.1074/jbc.M113.500397 PMC3829175

[55] Janin M, Davalos V, Esteller M. Cancer metastasis under the magnifying glass of epigenetics and epitranscriptomics. Cancer Metastasis Rev. 2023;42:1071–112. 10.1007/s10555-023-10120-3. 37369946 10.1007/s10555-023-10120-3 PMC10713773

[56] Kravitz CJ, Yan Q, Nguyen DX. Epigenetic markers and therapeutic targets for metastasis. Cancer Metastasis Rev. 2023;42:427–43. 10.1007/s10555-023-10109-y. 37286865 10.1007/s10555-023-10109-y PMC10595046

[57] Balacco DL, Soller M. The m ^6^ a writer: rise of a machine for growing tasks. Biochemistry. 2019;58:363–78. 10.1021/acs.biochem.8b01166. 30557013 10.1021/acs.biochem.8b01166

[58] Bawankar P, Lence T, Paolantoni C, Haussmann IU, Kazlauskiene M, Jacob D, Heidelberger JB, Richter FM, Nallasivan MP, Morin V, et al. Hakai is required for stabilization of core components of the m6A mRNA methylation machinery. Nat Commun. 2021;12:3778. 10.1038/s41467-021-23892-5. 34145251 10.1038/s41467-021-23892-5 PMC8213727

[59] Růžička K, Zhang M, Campilho A, Bodi Z, Kashif M, Saleh M, Eeckhout D, El-Showk S, Li H, Zhong S, et al. Identification of factors required for m ^6^ A mRNA methylation in *Arabidopsis* reveals a role for the conserved E3 ubiquitin ligase HAKAI. New Phytol. 2017;215:157–72. 10.1111/nph.14586. 28503769 10.1111/nph.14586 PMC5488176

[60] Hu J, Cai J, Park SJ, Lee K, Li Y, Chen Y, Yun J, Xu T, Kang H. *N*^6^ -Methyladenosine mRNA methylation is important for salt stress tolerance in *Arabidopsis*. Plant J. 2021;106:1759–75. 10.1111/tpj.15270. 33843075 10.1111/tpj.15270

[61] Furci L, Berthelier J, Saze H. RNA N6-adenine methylation dynamics impact *Hyaloperonospora arabidopsidis* resistance in *Arabidopsis*. Plant Physiol. 2024. 10.1093/plphys/kiae373. 38991559 10.1093/plphys/kiae373 PMC11812051

[62] Wang Y, Zhang L, Ren H, Ma L, Guo J, Mao D, Lu Z, Lu L, Yan D. Role of Hakai in m6A modification pathway in Drosophila. Nat Commun. 2021;12:2159. 10.1038/s41467-021-22424-5. 33846330 10.1038/s41467-021-22424-5 PMC8041851

[63] Zhang M, Bodi Z, Mackinnon K, Zhong S, Archer N, Mongan NP, Simpson GG, Fray RG. Two zinc finger proteins with functions in m6A writing interact with HAKAI. Nat Commun. 2022;13:1127. 10.1038/s41467-022-28753-3. 35236848 10.1038/s41467-022-28753-3 PMC8891334

[64] Yue Y, Liu J, Cui X, Cao J, Luo G, Zhang Z, Cheng T, Gao M, Shu X, Ma H, et al. VIRMA mediates preferential m6A mRNA methylation in 3′UTR and near stop codon and associates with alternative polyadenylation. Cell Discov. 2018;4:10. 10.1038/s41421-018-0019-0. 29507755 10.1038/s41421-018-0019-0 PMC5826926

[65] Schwartz S, Mumbach MR, Jovanovic M, Wang T, Maciag K, Bushkin GG, Mertins P, Ter-Ovanesyan D, Habib N, Cacchiarelli D, et al. Perturbation of m6A writers reveals two distinct classes of mRNA methylation at internal and 5′ sites. Cell Rep. 2014;8:284–96. 10.1016/j.celrep.2014.05.048. 24981863 10.1016/j.celrep.2014.05.048 PMC4142486

[66] Wen J, Lv R, Ma H, Shen H, He C, Wang J, Jiao F, Liu H, Yang P, Tan L, et al. Zc3h13 regulates nuclear RNA m6A methylation and mouse embryonic stem cell self-renewal. Mol Cell. 2018;69:1028-1038.e6. 10.1016/j.molcel.2018.02.015. 29547716 10.1016/j.molcel.2018.02.015 PMC5858226

[67] Hu C, Yang L, Wang Y, Zhou S, Luo J, Gu Y. Ginsenoside Rh2 reduces m6A RNA methylation in cancer via the KIF26B-SRF positive feedback loop. J Ginseng Res. 2021;45:734–43. 10.1016/j.jgr.2021.05.004. 34764728 10.1016/j.jgr.2021.05.004 PMC8569326

[68] Jiménez Martín O, Schlosser A, Furtwängler R, Wegert J, Gessler M. MYCN and MAX alterations in Wilms tumor and identification of novel N-MYC interaction partners as biomarker candidates. Cancer Cell Int. 2021;21:555. 10.1186/s12935-021-02259-2. 34689785 10.1186/s12935-021-02259-2 PMC8543820

[69] Wilson MM, Weinberg RA, Lees JA, Guen VJ. Emerging mechanisms by which EMT programs control stemness. Trends Cancer. 2020;6:775–80. 10.1016/j.trecan.2020.03.011. 32312682 10.1016/j.trecan.2020.03.011

[70] Dongre A, Weinberg RA. New insights into the mechanisms of epithelial–mesenchymal transition and implications for cancer. Nat Rev Mol Cell Biol. 2019;20:69–84. 10.1038/s41580-018-0080-4. 30459476 10.1038/s41580-018-0080-4

[71] Quiroga M, Rodríguez-Alonso A, Alfonsín G, Rodríguez JJE, Breijo SM, Chantada V, Figueroa A. Protein Degradation by E3 ubiquitin ligases in cancer stem cells. Cancers (Basel). 2022;14:990. 10.3390/cancers14040990. 35205738 10.3390/cancers14040990 PMC8870109

[72] Li N, Babaei-Jadidi R, Lorenzi F, Spencer-Dene B, Clarke P, Domingo E, Tulchinsky E, Vries RGJ, Kerr D, Pan Y, et al. An FBXW7-ZEB2 axis links EMT and tumour microenvironment to promote colorectal cancer stem cells and chemoresistance. Oncogenesis. 2019;8:13. 10.1038/s41389-019-0125-3. 30783098 10.1038/s41389-019-0125-3 PMC6381143

[73] Jeon S-A, Kim DW, Lee D-B, Cho J-Y. NEDD4 plays roles in the maintenance of breast cancer stem cell characteristics. Front Oncol. 2020. 10.3389/fonc.2020.01680. 33014839 10.3389/fonc.2020.01680 PMC7509455

[74] Chan C-H, Morrow JK, Li C-F, Gao Y, Jin G, Moten A, Stagg LJ, Ladbury JE, Cai Z, Xu D, et al. Pharmacological inactivation of Skp2 SCF ubiquitin ligase restricts cancer stem cell traits and cancer progression. Cell. 2013;154:556–68. 10.1016/j.cell.2013.06.048. 23911321 10.1016/j.cell.2013.06.048 PMC3845452

[75] Alfonsín G, Berral-González A, Rodríguez-Alonso A, Quiroga M, De Las RJ, Figueroa A. Stratification of colorectal patients based on survival analysis shows the value of consensus molecular subtypes and reveals the CBLL1 gene as a biomarker of CMS2 tumours. Int J Mol Sci. 2024;25:1919. 10.3390/ijms25031919. 38339195 10.3390/ijms25031919 PMC10856263

[76] Kaido M, Wada H, Shindo M, Hayashi S. Essential requirement for RING finger E3 ubiquitin ligase Hakai in early embryonic development of *Drosophila*. Genes Cells. 2009;14:1067–77. 10.1111/j.1365-2443.2009.01335.x. 19682089 10.1111/j.1365-2443.2009.01335.x

[77] Růžička K, Zhang M, Campilho A, Bodi Z, Kashif M, Saleh M, Eeckhout D, El-Showk S, Li H, Zhong S, et al. Identification of factors required for m6A mRNA methylation in Arabidopsis reveals a role for the conserved E3 ubiquitin ligase HAKAI. New Phytol. 2017;215:157–72. 10.1111/nph.14586. 28503769 10.1111/nph.14586 PMC5488176

[78] Przybyla L, Lakins JN, Weaver VM. Tissue mechanics orchestrate Wnt-dependent human embryonic stem cell differentiation. Cell Stem Cell. 2016;19:462–75. 10.1016/j.stem.2016.06.018. 27452175 10.1016/j.stem.2016.06.018 PMC5336327

[79] Jiang X, Liu B, Nie Z, Duan L, Xiong Q, Jin Z, Yang C, Chen Y. The role of m6A modification in the biological functions and diseases. Signal Transduct Target Ther. 2021;6:74. 10.1038/s41392-020-00450-x. 33611339 10.1038/s41392-020-00450-x PMC7897327

[80] Lan Y, Liu B, Guo H. The role of M6A modification in the regulation of tumor-related lncRNAs. Mol Ther Nucleic Acids. 2021;24:768–79. 10.1016/j.omtn.2021.04.002. 33996258 10.1016/j.omtn.2021.04.002 PMC8094576

[81] Song N, Cui K, Zhang K, Yang J, Liu J, Miao Z, Zhao F, Meng H, Chen L, Chen C, et al. The role of m6A RNA methylation in cancer: implication for nature products anti-cancer research. Front Pharmacol. 2022. 10.3389/fphar.2022.933332. 35784761 10.3389/fphar.2022.933332 PMC9243580

[82] Garcias Morales D, Reyes JL. A birds’-eye view of the activity and specificity of the mRNA m6A methyltransferase complex. WIREs RNA. 2021. 10.1002/wrna.1618. 32686365 10.1002/wrna.1618

[83] Horiuchi K, Kawamura T, Hamakubo T. Wilms’ tumor 1-associating protein complex regulates alternative splicing and polyadenylation at potential G-quadruplex-forming splice site sequences. J Biol Chem. 2021;297: 101248. 10.1016/j.jbc.2021.101248. 34582888 10.1016/j.jbc.2021.101248 PMC8605363

[84] Fang R, Ye L, Shi H. Understanding the roles of N6-methyladenosine writers, readers and erasers in breast cancer. Neoplasia. 2021;23:551–60. 10.1016/j.neo.2021.04.002. 34000587 10.1016/j.neo.2021.04.002 PMC8138681

[85] Wang N, Xu Y, Jin L, Wang X, Wu S, Wang Y, Zhao J, Zhou F, Ge H. Effects of N6-methyladenosine regulators on LAG3 and immune infiltrates in lung adenocarcinoma. Dis Markers. 2022;2022:1–24. 10.1155/2022/1829528. 10.1155/2022/1829528 PMC9427291 36051357

[86] Guinney J, Dienstmann R, Wang X, de Reyniès A, Schlicker A, Soneson C, Marisa L, Roepman P, Nyamundanda G, Angelino P, et al. The consensus molecular subtypes of colorectal cancer. Nat Med. 2015;21:1350–6. 10.1038/nm.3967. 26457759 10.1038/nm.3967 PMC4636487

[87] Zhou W-J, Geng ZH, Chi S, Zhang W, Niu X-F, Lan S-J, Ma L, Yang X, Wang L-J, Ding Y-Q, et al. Slit-Robo signaling induces malignant transformation through Hakai-mediated E-cadherin degradation during colorectal epithelial cell carcinogenesis. Cell Res. 2011;21:609–26. 10.1038/cr.2011.17. 21283129 10.1038/cr.2011.17 PMC3203654

[88] Roca-Lema D, Quiroga M, Khare V, Díaz-Díaz A, Barreiro-Alonso A, Rodríguez-Alonso A, Concha Á, Romay G, Cerdán ME, Gasche C, et al. Role of the E3 ubiquitin ligase Hakai in intestinal inflammation and cancer bowel disease. Sci Rep. 2022;12:17571. 10.1038/s41598-022-22295-w. 36266428 10.1038/s41598-022-22295-w PMC9584894

[89] Hou Z, Peng H, Ayyanathan K, Yan K-P, Langer EM, Longmore GD, Rauscher FJ. The LIM protein AJUBA recruits protein arginine methyltransferase 5 to mediate SNAIL-dependent transcriptional repression. Mol Cell Biol. 2008;28:3198–207. 10.1128/mcb.01435-07. 18347060 10.1128/MCB.01435-07 PMC2423142

[90] Chen Y, Chen C-H, Tung P-Y, Huang S-H, Wang S-M. An acidic extracellular pH disrupts adherens junctions in HepG2 cells by Src kinases-dependent modification of E-cadherin. J Cell Biochem. 2009;108:851–9. 10.1002/jcb.22313. 19711372 10.1002/jcb.22313

[91] Liu Z, Wu Y, Tao Z, Ma L. E3 ubiquitin ligase Hakai regulates cell growth and invasion, and increases the chemosensitivity to cisplatin in non-small-cell lung cancer cells. Int J Mol Med. 2018;42:1145–51. 10.3892/ijmm.2018.3683. 29786107 10.3892/ijmm.2018.3683

[92] Suda K, Rozeboom L, Rivard CJ, Yu H, Ellison K, Melnick MAC, Hinz TK, Chan D, Heasley LE, Politi K, et al. Therapy-induced E-cadherin downregulation alters expression of programmed death ligand-1 in lung cancer cells. Lung Cancer. 2017;109:1–8. 10.1016/j.lungcan.2017.04.010. 28577937 10.1016/j.lungcan.2017.04.010 PMC6174882

[93] Weng C-H, Chen L-Y, Lin Y-C, Shih J-Y, Lin Y-C, Tseng R-Y, Chiu A-C, Yeh Y-H, Liu C, Lin Y-T, et al. Epithelial-mesenchymal transition (EMT) beyond EGFR mutations per se is a common mechanism for acquired resistance to EGFR TKI. Oncogene. 2019;38:455–68. 10.1038/s41388-018-0454-2. 30111817 10.1038/s41388-018-0454-2

[94] Chu I, Arnaout A, Loiseau S, Sun J, Seth A, McMahon C, Chun K, Hennessy B, Mills GB, Nawaz Z, et al. Src promotes estrogen-dependent estrogen receptor α proteolysis in human breast cancer. J Clin Investig. 2007;117:2205–15. 10.1172/JCI21739. 17627304 10.1172/JCI21739 PMC1906730

[95] Gong E-Y, Park E, Lee K. Hakai acts as a coregulator of estrogen receptor alpha in breast cancer cells. Cancer Sci. 2010;101:2019–25. 10.1111/j.1349-7006.2010.01636.x. 20608937 10.1111/j.1349-7006.2010.01636.x PMC11159366

[96] Zheng F, Du F, Qian H, Zhao J, Wang X, Yue J, Hu N, Si Y, Xu B, Yuan P. Expression and clinical prognostic value of m6A RNA methylation modification in breast cancer. Biomark Res. 2021;9:28. 10.1186/s40364-021-00285-w. 33926554 10.1186/s40364-021-00285-w PMC8082898

[97] Martinez-Iglesias O, Casas-Pais A, Castosa R, Díaz-Díaz A, Roca-Lema D, Concha Á, Cortés Á, Gago F, Figueroa A. Hakin-1, a new specific small-molecule inhibitor for the E3 ubiquitin ligase Hakai, inhibits carcinoma growth and progression. Cancers (Basel). 2020;12:1340. 10.3390/cancers12051340. 32456234 10.3390/cancers12051340 PMC7281109

[98] Liu Z, Fan M, Xuan X, Xia C, Huang G, Ma L. Celastrol inhibits the migration and invasion and enhances the anti-cancer effects of docetaxel in human triple-negative breast cancer cells. Med Oncol. 2022;39:189. 10.1007/s12032-022-01792-y. 36071249 10.1007/s12032-022-01792-y

[99] Aparicio LA, Castosa R, Haz-Conde M, Rodríguez M, Blanco M, Valladares M, Figueroa A. Role of the microtubule-targeting drug vinflunine on cell-cell adhesions in bladder epithelial tumour cells. BMC Cancer. 2014. 10.1186/1471-2407-14-507. 25012153 10.1186/1471-2407-14-507 PMC4107965

[100] Deep G, Gangar SC, Agarwal C, Agarwal R. Role of E-cadherin in antimigratory and antiinvasive efficacy of silibinin in prostate cancer cells. Cancer Prev Res. 2011;4:1222–32. 10.1158/1940-6207.CAPR-10-0370. 10.1158/1940-6207.CAPR-10-0370 PMC3151351 21546539

[101] Gu Z, Du Y, Zhao X, Wang C. Diagnostic, therapeutic, and prognostic value of the m6a writer complex in hepatocellular carcinoma. Front Cell Dev Biol. 2022. 10.3389/fcell.2022.822011. 35223847 10.3389/fcell.2022.822011 PMC8864226

[102] Yang Y, Qian Z, Feng M, Liao W, Wu Q, Wen F, Li Q. Study on the prognosis, immune and drug resistance of m6A-related genes in lung cancer. BMC Bioinformatics. 2022;23:437. 10.1186/s12859-022-04984-5. 36261786 10.1186/s12859-022-04984-5 PMC9583491

[103] Zhu M, Cui Y, Mo Q, Zhang J, Zhao T, Xu Y, Wu Z, Sun D, Zhang X, Li Y, et al. Characterization of m6A RNA methylation regulators predicts survival and immunotherapy in lung adenocarcinoma. Front Immunol. 2021. 10.3389/fimmu.2021.782551. 34975871 10.3389/fimmu.2021.782551 PMC8718692

[104] Guo X, Zhang Y, Zheng L, Zheng C, Song J, Zhang Q, Kang B, Liu Z, Jin L, Xing R, et al. Global characterization of T cells in non-small-cell lung cancer by single-cell sequencing. Nat Med. 2018;24:978–85. 10.1038/s41591-018-0045-3. 29942094 10.1038/s41591-018-0045-3

[105] Zhou B, Gao S. Comprehensive analysis of clinical significance, immune infiltration and biological role of m6A regulators in early-stage lung adenocarcinoma. Front Immunol. 2021. 10.3389/fimmu.2021.698236. 34650549 10.3389/fimmu.2021.698236 PMC8505809

[106] Zhao H, Xu Y, Xie Y, Zhang L, Gao M, Li S, Wang F. m6A regulators is differently expressed and correlated with immune response of esophageal cancer. Front Cell Dev Biol. 2021. 10.3389/fcell.2021.650023. 33748145 10.3389/fcell.2021.650023 PMC7970005

[107] Zhang C, Gu L, Xiao J, Jin F. Knockdown of RBM15 inhibits tumor progression and the JAK-STAT signaling pathway in cervical cancer. BMC Cancer. 2023;23:684. 10.1186/s12885-023-11163-z. 37474926 10.1186/s12885-023-11163-z PMC10360283

[108] Su H, Wang Y, Li H. RNA m6A methylation regulators multi-omics analysis in prostate cancer. Front Genet. 2021. 10.3389/fgene.2021.768041. 34899855 10.3389/fgene.2021.768041 PMC8661905

[109] Zhang Y, Wang X, Duan X, Du T, Chen X. The synergistic effect of EMT regulators and m6A modification on prognosis-related immunological signatures for ovarian cancer. Sci Rep. 2023;13:14872. 10.1038/s41598-023-41554-y. 37684273 10.1038/s41598-023-41554-y PMC10491820

[110] Tan W, Liu S, Deng Z, Dai F, Yuan M, Hu W, Li B, Cheng Y. Gene signature of m6A-related targets to predict prognosis and immunotherapy response in ovarian cancer. J Cancer Res Clin Oncol. 2023;149:593–608. 10.1007/s00432-022-04162-3. 36048273 10.1007/s00432-022-04162-3 PMC11797572

[111] Crystal structure of a phosphotyrosine binding domain. 2012; 10.2210/pdb3vk6/pdb

[112] Structure of native c-Cbl. 2012; 10.2210/pdb2y1m/pdb

[113] Dou H, Buetow L, Hock A, Sibbet GJ, Vousden KH, Huang DT. Structural basis for autoinhibition and phosphorylation-dependent activation of c-Cbl. Nat Struct Mol Biol. 2012;19:184–92. 10.1038/nsmb.2231. 22266821 10.1038/nsmb.2231 PMC3880865

[114] Berman HM. The Protein Data Bank. Nucleic Acids Res. 2000;28:235–42. 10.1093/nar/28.1.235. 10592235 10.1093/nar/28.1.235 PMC102472

